# Mortality and its predictors among human immunodeficiency virus-infected children younger than 15 years receiving antiretroviral therapy in Ethiopia: a systematic review and meta-analysis

**DOI:** 10.1186/s12879-024-09366-1

**Published:** 2024-05-03

**Authors:** Beshada Zerfu Woldegeorgis, Yordanos sisay Asgedom, Amanuel Yosef Gebrekidan, Gizachew Ambaw Kassie, Ushula Deboch Borko, Mohammed Suleiman Obsa

**Affiliations:** 1 Department of Internal Medicine, College of Health Sciences and Medicine, Wolaita Sodo University, Wolaita Sodo, Ethiopia; 2 Department of Epidemiology, College of Health Sciences and Medicine, Wolaita Sodo University, Wolaita Sodo, Ethiopia; 3 School of Public Health, College of Health Sciences and Medicine, Wolaita Sodo University, Wolaita Sodo, Ethiopia; 4 Department of Anesthesia, Arsi University, Asella, Ethiopia

**Keywords:** Children, Meta-analysis, HIV/AIDS, Antiretroviral therapy, Predictors, Survival, Ethiopia

## Abstract

**Background:**

Despite antiretroviral treatment (ART), the human immunodeficiency virus (HIV) continues to pose a considerable health burden in resource-poor countries. This systematic review and meta-analysis aimed to determine the pooled incidence density of mortality and identify potential predictors among HIV-infected children receiving ART, from studies conducted in various parts of Ethiopia.

**Methods:**

A comprehensive database search was made in Excerpta Medica, PubMed, Web of Science, African Journals Online, Google Scholar, and Scopus. We reported results following the Preferred Reporting Items for Systematic Reviews and Meta-Analysis 2020. Excel Spreadsheet and STATA Version 14 software were used for data abstraction and meta-analysis, respectively. Statistical heterogeneity among studies was assessed using *I*^*2*^ statistics. Meta-regression and subgroup analysis were performed to further explore the sources of statistical heterogeneity. Moreover, publication bias and a leave-out-one sensitivity analysis were performed.

**Results:**

Twenty-two articles involving 8,731 participants met inclusion criteria and were included. The pooled incidence density of mortality was 3.08 (95% confidence interval (CI), 2.52 to 3.64) per 100 child years. Predictors of mortality were living in rural areas (hazard ratio (HR), 2.18 [95% CI, 1.20 to 3.98]), poor adherence to ART (HR, 2.85 [ 95% CI, 1.39 to 5.88]), failure to initiate co-trimoxazole preventive therapy (HR, 2.16 [95% CI, 1.52 to 3.07]), anemia (HR, 2.28 [95% CI, 1.51 to 3.45]), opportunistic infections (HR, 1.52 [ 95% CI, 1.15 to 2.00]), underweight (HR, 1.74 [95% CI, 1.26 to 2.41]), wasting (HR, 2.54 [95% CI, 1.56 to 4.16]), stunting (HR, 2.02 [95% CI, 1.63 to 2.51]), World Health Organization classified HIV clinical stages III and IV (HR, 1.71 [95% CI, 1.42 to 2.05]), and Nevirapine-based regimens (HR, 3.91 [95% CI, 3.09 to 4.95]).

**Conclusions:**

This study found that the overall mortality rate among HIV-infected children after ART initiation was high. Therefore, high-level commitment and involvement of responsible caregivers, healthcare providers, social workers, and program managers are of paramount importance to identify these risk factors and thus enhance the survival of HIV-infected children receiving ART.

**Supplementary Information:**

The online version contains supplementary material available at 10.1186/s12879-024-09366-1.

## Background

By the end of 2022, an estimated 39 million people were living with the human immunodeficiency virus (HIV) (1.5 million of whom were children younger than 15 years of age), and an estimated 630,000 people died from HIV-related causes [1]. Besides, estimates from the World Health Organization (WHO) suggest that an estimated 29.8 million persons living with the HIV worldwide (57% of whom were children younger than 15 years) were receiving antiretroviral therapy (ART) in the same period [2].

In Ethiopia, children younger than 15 years of age make up 39% of the general population (an estimated 49.3 million) [3]; an estimated 36,812 children were living with HIV, and 1,483 deaths were recorded in those receiving ART [4]. Owing to the introduction and scaling-up of ART, HIV-related mortality has steadily declined over the past two decades in developed countries, however, the problem remains important in low-resource settings including Ethiopia [1, 5]. A meta-analysis study revealed that the cumulative incidence of death among HIV-infected children after initiating ART was estimated to be 8% in sub-Saharan African countries [6], which ascertains that HIV treatment is still a challenge in low-resource settings.

Given that HIV infection is chronic and treatment is lifelong, it is worthwhile to assess the overall rates and predictors of death among children following ART initiation to enhance long-term survival. In Ethiopia, cohort studies conducted among HIV-infected children initiating ART have revealed significantly wide geographic variations of mortality with the incidence of 1.08 deaths per 100 child years [7] to 12.94 deaths per 100 child years [8]. Furthermore, evidence on patient demographics and nutritional status, baseline laboratory, treatment, and other clinical conditions is of paramount importance for providing comprehensive HIV care, improving the survival of children receiving ART, and being a springboard for decision-making in HIV-related programs and health policies in Ethiopia. Therefore, this systematic review and meta-analysis aimed to answer the following two major questions: (1) What is the pooled incidence density of mortality among HIV-infected children after ART initiation in Ethiopia? (2) What are the predictors of mortality among HIV-infected children after ART initiation in Ethiopia?

## Methods

### Registration and reporting

The protocol to conduct this study was developed following the Preferred Reporting Items for Systematic Reviews and Meta-Analysis (PRISMA)-Protocol 2015 guideline [9] and it was registered in PROSPERO with the registration number CRD42023445028. Findings were reported following the PRISMA-2020 statement [10] (see Additional file 1).

### Inclusion and exclusion criteria

To determine whether a study was eligible for inclusion in the review, the components described in the CoCoPop (Co = condition/domain; Co = context/settings; Pop = population/participants) framework [11] were used. The population/participants were HIV-infected children younger than 15 years of age who received ART in Ethiopia. Condition/ domain: Studies that properly identified and defined the factors of interest based on the incidence and/or predictors of mortality were considered. Settings/context: Observational epidemiological studies restricted to Ethiopia are considered. In addition, articles published in English from inception to August 31, 2023, were eligible for inclusion in the review. In contrast, studies without access to full text, articles that did not measure or contained insufficient information on mortality and/or predictor variables, personal opinions, qualitative study design, case reports, review articles, case series, conference abstracts, letters to editors, commentaries, and unpublished data were ineligible.

### Information sources and search strategy

To ensure complete coverage of the topic by accounting for variability in the indexing in each database, a double-blinded search by BZW and YSA was conducted in the electronic bibliographic medical databases of Excerpta Medica, PubMed, Web of Science, African Journals Online, Google Scholar, and Scopus from March 1, 2023, to August 31, 2023. Moreover, the chain referral procedure method was applied to address the literature saturation. For the PubMed and related databases, relevant search terms were collected after conducting a preliminary search in Google Scholar, Wikipedia, title and keywords, and Google for each concept, and then combined in an advanced search using Boolean logic (“AND” and “OR”), double quotes, and truncation. The search terms employed include: “ mortality”, “death”, “survival”, “HIV/AIDS”, “Human immune deficiency virus”, “acquired immune deficiency syndrome”, “ART”, “antiretroviral therapy”, “HAART”, “highly active antiretroviral therapy”, “prevalence,” “proportion”, “incidence”, “associated factors”, “predictors”, “determinants”, “child*”, “pediatrics”, “paediatrics”, “Ethiopia” (see Additional file 2 ).

### Study selection and data extraction

The articles were exported to EndNote X7, where duplicate ones were then removed. MSO and BZW screened the titles and abstracts independently. AYK and GAK conducted a full article review against the predefined criteria. Where additional information was required to answer queries regarding eligibility, other authors were involved as needed. Disagreements about whether a study should be included were resolved by discussion among each other. Regarding data extraction, two authors (BZW and UDB) working independently, excerpted the relevant data by using a standardized Microsoft Excel spreadsheet 2010. The abstraction format captured data on the following major components: first author, publication time, study design, number of participants, study settings, number of deaths, observation period, person-time at risk, death rates per 100 child years, median follow-up time, median survival, and the response rate.

### Methodological quality assessment

Two authors (BZW and MSO) evaluated the original studies according to the Joanna Briggs Institute critical appraisal checklist for cohort study (https://joannabriggs.org/critical_appraisal_tools*)* which included 11 constructs. The response options were labeled as ‘yes’, ‘no’, and ‘unclear question’. The total score was computed by counting the number of ‘yes’ answers in each row. Articles with critical appraisal scores of 7 and above were included in the systematic review and meta-analysis (see Additional file 3 ).

### Outcome and effect measures

The primary outcome of this systematic review and meta-analysis was the incidence of mortality in HIV-infected children aged < 15 years. The incidence density of mortality was calculated by dividing the number of deaths by the person-time at risk and multiplying by 100. The appropriate effect measure for our study was the hazard ratio (HR). We categorized the predictor variables as follows: residence (rural vs. urban), ART adherence (poor vs. good and fair), co-trimoxazole preventive therapy (CPT) (yes vs. no), isoniazid preventive therapy (IPT) (yes vs. no), hemoglobin (< 10 g/dl vs. ≥ 10 g/dl), opportunistic infections (OIs) (yes vs. no), weight for age (underweight vs. normal), weight for height (wasted vs. normal), height for age (stunted vs. normal), the WHO classified HIV clinical stages (III and IV vs. I and III), cluster of differentiation 4 (CD4) + T lymphocyte count (below threshold vs. normal), Nevirapine (NVP)-based regimens (yes vs. no) and treatment failure (yes vs. no).

### Synthesis methods

The meta-analysis was conducted using STATA Version 14 [12]. We employed the random-effect meta-analysis model to estimate the Der Simonian and Laird’s pooled effect, as considerable statistical heterogeneity was observed in the fixed-effect meta-analysis model. The presence and degree of between-study (heterogeneity) variance among individual studies were evaluated graphically via forest plots and formally via a statistical test for variance (the chi-squared test, significance level ≥ 0.1; *I*^2^:0–25%: low heterogeneity; 25–50%: moderate heterogeneity; 50–75%: high heterogeneity; 75–100%: very high heterogeneity) [13]. Subgroup analysis (based on publication period, sample size, and region) and meta-regression (based on publication period and sample size) were performed to explore source of statistical heterogeneity. The sensitivity analysis was also performed. Reporting bias was explored using the Egger test (significant at *P* < 0.05) and funnel plots [14]. Due to the presence of reporting bias for the pooled incidence of mortality, we used the non-parametric trim-and-fill method of Duval and Tweedie. Variables with *P* ≤ 0.05 were deemed statistically significant predictors of mortality, and the strength of the association was presented by HR with a corresponding 95% confidence interval (CI).

## Results

### Study selection

**Fig. 1:**
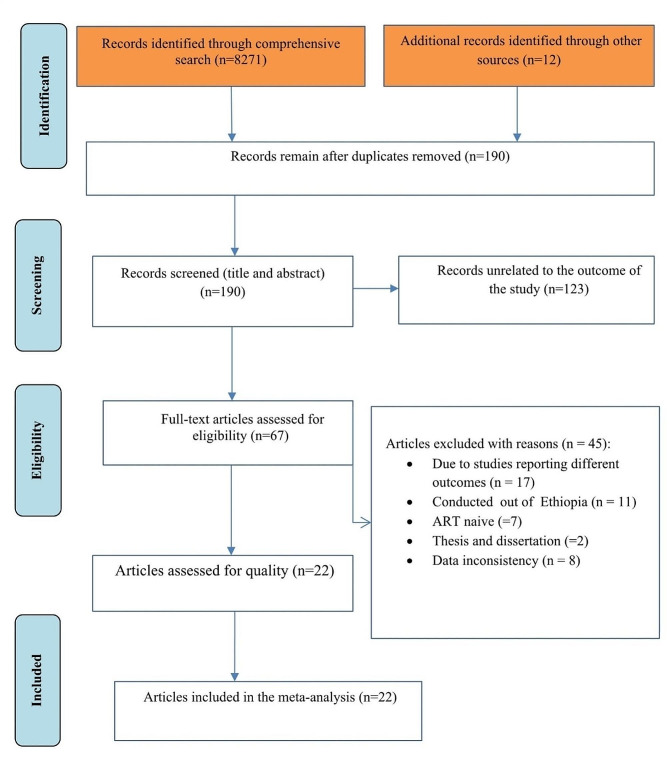
Flow diagram of articles selection and screening process

A comprehensive medical database search identified 8,283 records. After duplicates were removed, 190 remained and were screened based on their titles and abstracts, with 123 removed as unrelated to the study domain. Sixty-seven full-text articles were evaluated against the inclusion criteria, of which 45 were excluded. Ultimately, 22 articles were included in the meta-analysis (Fig. 1).

### Study characteristics

Twenty-two articles [7, 8, 15–34] involving 8,731 children younger than 15 years were included. 51% (*n* = 4406) of the study participants were male. All studies employed a retrospective cohort epidemiological study design and reported data were abstracted from hospitals and health centers’ ART databases, patient records, and follow-up forms. The original articles’ publication period ranged from 2011 [22] to 2022 [7, 24, 27, 31]. Based on administrative regions of Ethiopia, two studies were conducted in Oromia [16, 19], nine in Amhara [7, 8, 18, 21, 26, 30–32, 35], four in Addis Ababa, the nation’s capital [15, 22, 23, 28], four in the Southern Nations, Nationalities and Peoples Region (SNNPR) [20, 27, 29, 33], two in Tigray [24, 26], and one in Harari regions [17]. The sample ranges from 228 [29] to 757 [29]. The minimum and maximum person-time of observation were 329 child-years [22] and 4112 child-years [15], respectively. Individual study estimates of mortality range from 1.08 per 100 child years [7] to 12.94 per 100 child years [8]. The overall appraisal scores range from 9 to 11, indicating that the quality of the included articles in the meta-analysis was high (Table 1).

**Table 1:** Descriptive characteristics of studies included in the systematic review and meta-analysis

First author, year of publication	Settings/health facilities	Region	Study design	Follow-up period	Sample size	Person time at risk	Number of deaths	Death rates per 100 child years	Median follow-up	Response rate	Score
Mulugeta, 2017 [15]	St. Paul Referral Hospital and Millennium Medical College, St. Peter Referral Hospital, Zewuditu Memorial Hospital, and Yekatit 12 Hospital	Addis Ababa	Retrospective cohort	2008–2015	757	4112	51	1.24	68 months (IQR,62,83)	100%	10
Gesesew, 2018 [16]	Jimma University Teaching Hospital	Oromia	Retrospective cohort	2003–2015	399	1171.17	26	2.22	49 months	100%	10
Edessa, 2016 [17]	Hiwot Fana Specialised University Hospital and Jugol General Hospital	Harari	Retrospective cohort	2010–2013	305	609	28	4.60	30 months (IQR,18,30)	100%	11
Chanie, 2021 [18]	Debre Tabor Compressive Specialized Hospital, Gondar Compressive Specialized Hospital, and South Gondar Primary Hospitals	Amhara	Retrospective cohort	2014–2021	239	1063.17	39	3.67	Not reported	100%	10
Kedir, 2014 [19]	Adama Referral Hospital and Medical College	Oromia	Retrospective cohort	2006–2013	560	2078	43	2.10	47 months (IQR,29, 62)	100%	11
Atnafu, 2012 [8]	Felege-Hiwot Referral Hospital	Amhara	Retrospective cohort	2007–2009	255	548.75	71	12.94	Not reported	100%	11
Dawit, 2021 [20]	seven selected public hospitals in Southern Ethiopia	SNNPR	Retrospective cohort	2009–2018	274	1581.3	47	2.97	72 months (IQR, 36,96)	95.81%	11
Alebel, 2020 [21]	Debre Markos Referral Hospital, Felege Hiwot Comprehensive Specialized Hospital, and University of Gondar Comprehensive Specialized Hospital	Amhara	Retrospective cohort	2012–2017	538	1216.7	38	3.12	Not reported	97.29%	10
Asfawesen, 2011 [22]	All African Leprosy and Rehabilitation Centre	Addis Ababa	Retrospective cohort	2005–2008	482	329	13	3.95	16 months (IQR, 6,24)	100%	10
Ebissa, 2015 [23]	Black Lion Hospital, Yekatit 12 Hospital, Zewditu Hospital, and ALERT Hospital	Addis Ababa	Retrospective cohort	2008–2009	556	1054	58	5.50	Not reported	100%	9
Nigussie, 2022 [24]	Mekelle Hospital, Alamata Hospital and Maychew Hospital	Tigray	Retrospective cohort	2008–2018	253	2574	38	1.48	Not reported	89.7%	10
Koye, 2012 [25]	Felege Hiwot Referral Hospital	Amhara	Retrospective cohort	2006–2011	549	2025	41	4.00	22 months (IQR,28,62)	100%	10
Gebremedhin, 2013 [26]	Mekelle Hospital	Tigray	Retrospective cohort	2006–2011	416	1186.25	20	1.69	36 months	96.4%	10
Gemechu, 2022 [27]	Hawasa University Referral Hospital, Wolaita Sodo University Teaching Referral Hospital, Nigist Eleni Mohammad Memorial Referral Hospital, Yirgalem General Hospital, Arba Minch General Hospital, and Jinka General Hospital	SNNPR	Retrospective cohort	2009–2019	284	1257.17	35	2.78	54 months	87.4%	10
Tagesse, 2020 [28]	Tikur Anbessa Specialized Hospital	Addis Ababa	Retrospective cohort	2011–2015	410	1103	22	1.20	36 months (IQR,18,44)	100%	10
Bitew, 2017 [29]	Three hospitals, 68 governmental health centers, and two non-governmental health centers in the Wolaita Zone	SNNPR	Retrospective cohort	2006–2014	228	761.9	16	2.10	40 months(IQR,1,97 )	87.7%	11
Atalell, 2018 [30]	University of Gondar Comprehensive Specialized Hospital.	Amhara	Retrospective cohort	2005–2017	271	1167.67	38	3.25	48 months (IQR,22.8,78)	100%	10
Biyazin, 2022 [31]	Debre Markos Referral Hospital and Shegaw Mota District Hospital	Amhara	Retrospective cohort	2014–2018	251	626	16	2.56	Not reported	100%	10
Arage, 2019 [32]	Debre Tabor General Hospital and Dessie Referral Hospital	Amhara	Retrospective cohort	2005–2015	426	1548.6	97	6.26	44 months	92.2%	10
Chekole, 2022 [7]	Bahir Dar city public health facilities	Amhara	Retrospective cohort	2010–2019	588	2505.19	27	1.08	51 months	100%	10
Sidamo, 2018 [33]	Arba Minch Hospital and Arba Minch Health Center	SNNPR	Retrospective cohort	2009–2016	421	2719.5	65	2.39	50 months (IQR,24,80)	100%	10
Andargie, 2018 [34]	University of Gondar Comprehensive Specialized Hospital	Amhara	Retrospective cohort	2008–2015	269	1476	46	3.12	Not reported	100%	10

*Abbreviations* IQR, Interquartile range; SNNPR, the Southern Nations, Nationalities and Peoples Region

### Mortality

**Fig. 2:**
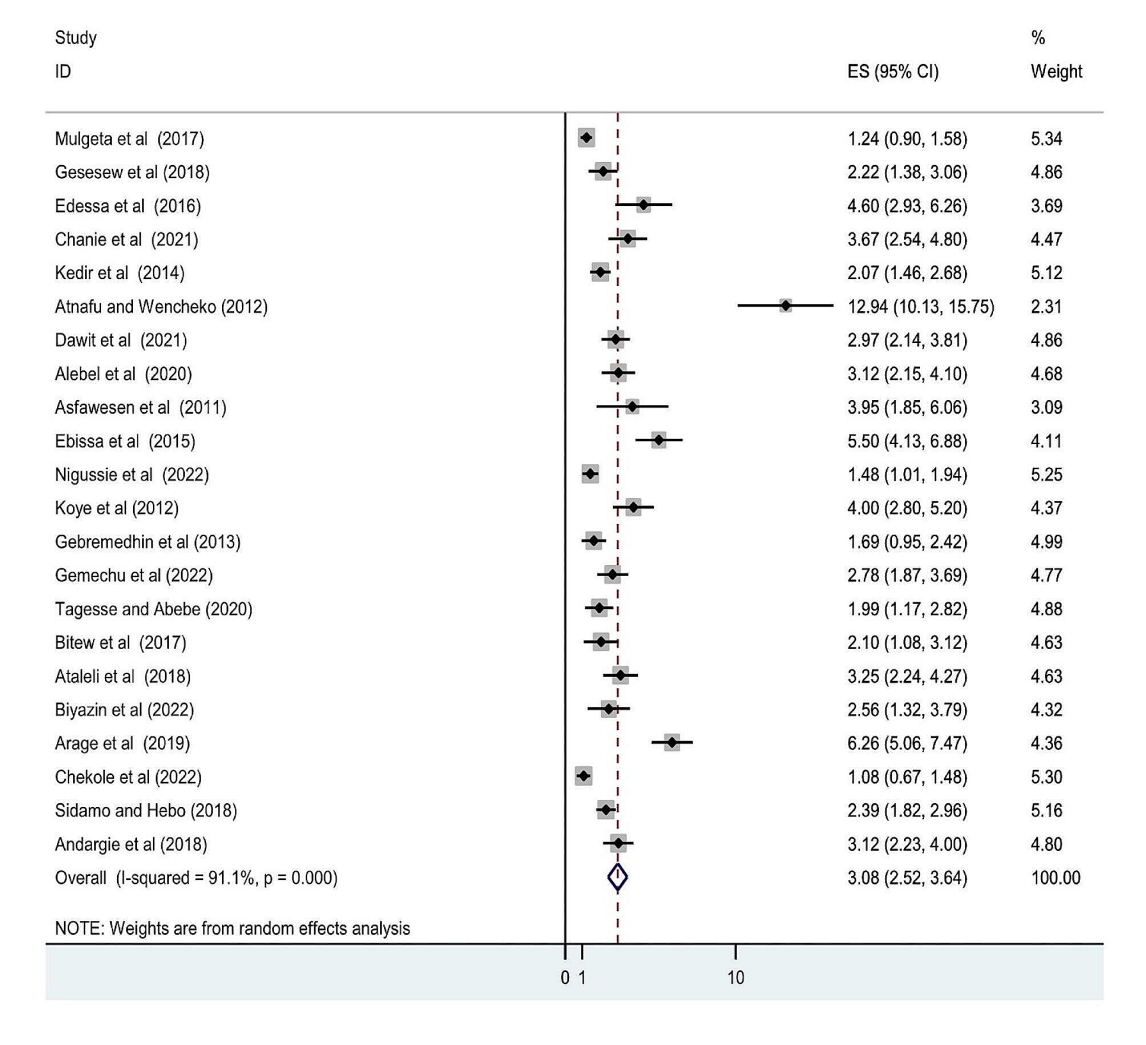
Forest plots of the pooled mortality proportion among HIV-infected children after ART initiation in Ethiopia

We analyzed the statistical heterogeneity of the 22 retrospective follow-up studies (*I*^*2*^ = 91.1% and *P* < 0.001). The weighted random effect meta-analysis revealed that the pooled incidence density of mortality among HIV-infected children after ART initiation was 3.08 (95% CI, 25.2 to 36.4) per 100 child years in Ethiopia (Fig. 2).

### Subgroup analysis and meta-regression

A subgroup meta-analysis was performed using sample size, period of publication, and administrative region of Ethiopia. Statistical heterogeneity ranged from 0.0 to 94.8%. The sub-group analysis revealed that mortality was 4.60 (95% CI, 2.93 to 6.26) per 100 child years in Harari region, 3.26 (95% CI, 2.57 to 3.95) per 100 child years when sample size was less than 500, and 3.08 (95% CI, 2.52 to 3.64) per 100 child years in articles published in 2016 and after (Table 2). Furthermore, meta-regression was performed, and the results revealed that while there was a significant difference in the year of publication (t = -2.37, *P* = 0.029), the sample size was insignificant (t = -1.71, *P* = 0.104).

**Table 2:** A subgroup analysis of mortality among HIV-infected children after ART initiation

Subgroups comparison	Reference(s)	Number of studies	Heterogeneity		Mortality per 100 child years (95% confidence interval )
			I^2^	*P* .value	
Regions/City administrations					
Oromia	[16, 19]	2	0.0%	0.777	2.12 (1.63, 2.62)
Amhara	[7, 8, 18, 21, 26, 30–32, 35]	9	94.8%	< 0.001	4.15 (2.73, 5.50)
Tigray	[24, 26]	2	0.0%	0.636	1.54 (1.14, 1.93)
Addis Ababa	[15, 22, 23, 28]	4	92.7%	< 0.001	3.03 (1.32, 4.73)
SNNPR	[20, 27, 29, 33]	4	0.0%	0.516	2.55 (2.16, 2.93)
Harari	[17]	1	-	-	4.60 (2.93, 6.26)
Sample size					
< 500	[8, 16–18, 20, 22, 24, 26–34]	16	88.8%	< 0.001	3.26 ( 2.57, 3.95)
≥ 500	[7, 15, 19, 21, 23, 25]	6	92.7%	< 0.001	2.65 (1.70, 3.61 )
Publication period					
Before 2016	[8, 19, 22, 23, 25, 26]	6	94.1%	< 0.001	3.08 (2.52, 3.64)
2016 and after	[7, 15–18, 20, 21, 24, 27–34]	16	89.1%	< 0.001	2.69 ( 2.14, 3.25)

*Note* SNNPR, Southern Nations, Nationalities and Peoples Region

### Sensitivity analysis

**Fig. 3:**
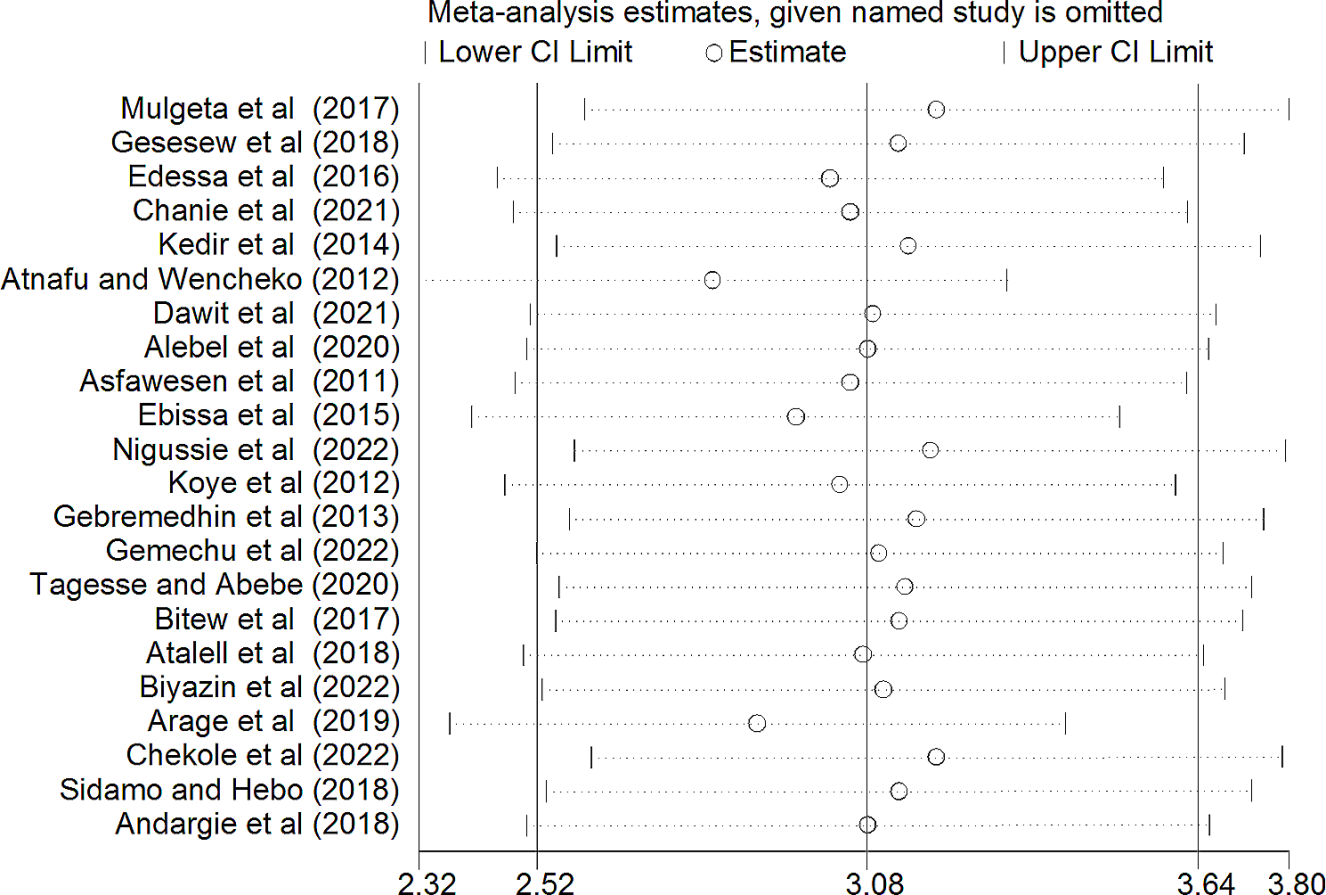
A leave-out one sensitivity analysis

A leave-out-one sensitivity analysis was performed to assess the impact of each study on the pooled rate of mortality while gradually excluding each study. Results showed that the combined effects did not change significantly (Fig. 3).

### Publication bias

**Fig. 4:**
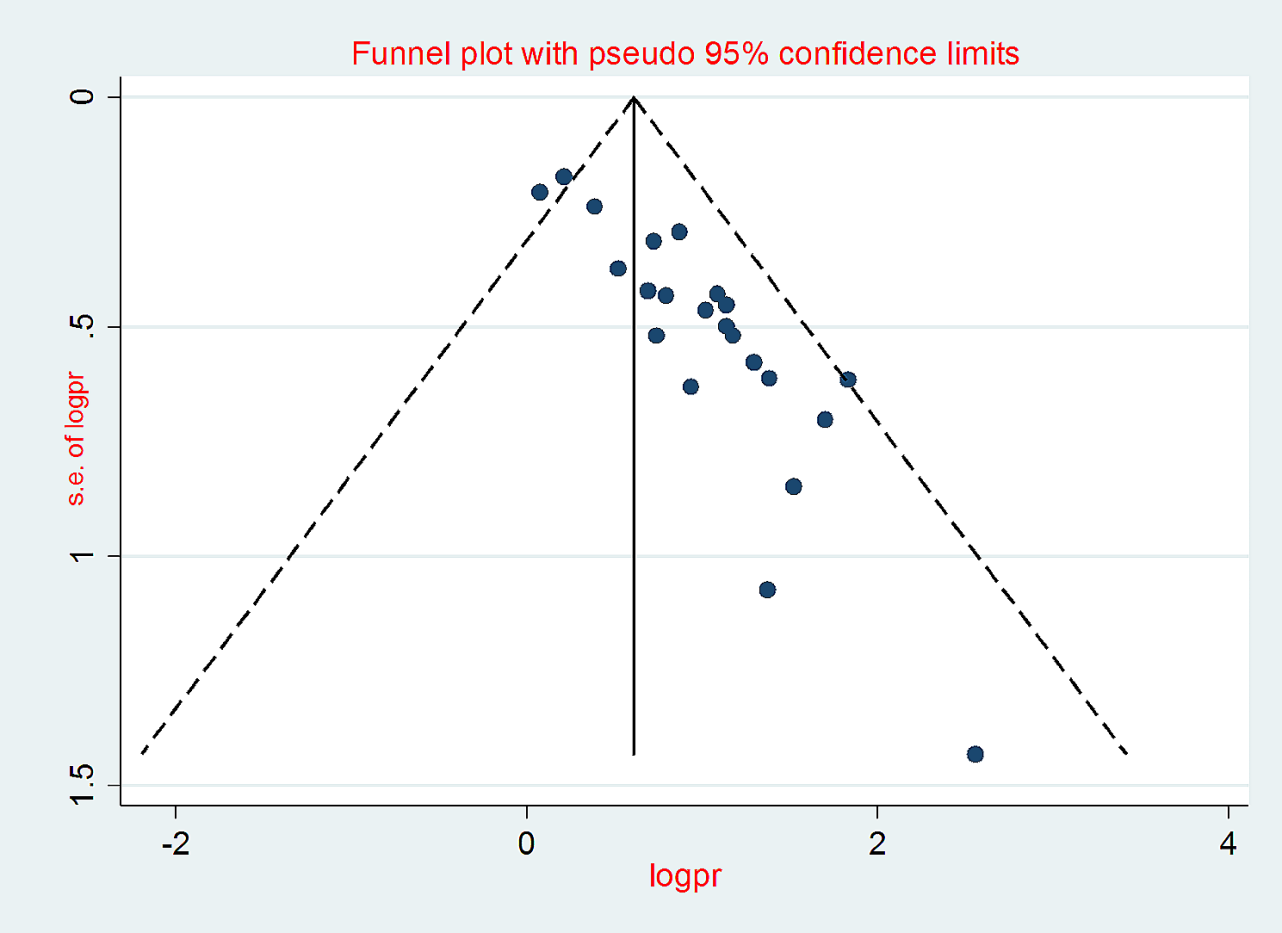
Funnel plots of the pooled mortality proportion among HIV infected children after ART initiation in Ethiopia

At a *P*-value of < 0.05, both Egger’s and Begg’s tests for small-study effects plots revealed publication biases. The funnel plots of the pooled incidence density of mortality among HIV-infected children after initiation of ART in Ethiopia showed significant asymmetry, on visual inspection (Fig. 4) and the combination of the results of the regression-based test of Egger ( t = 8.52, *P* < 0.001), and the nonparametric rank correlation test of Begg ( Z = 3.52, *P* < 0.001) showed the presence of evidence of small study effects. When evaluated against the Egger© regression test the P value was < 0.001. The test thus provides strong evidence for the presence of a small study effect.

**Fig. 5:**
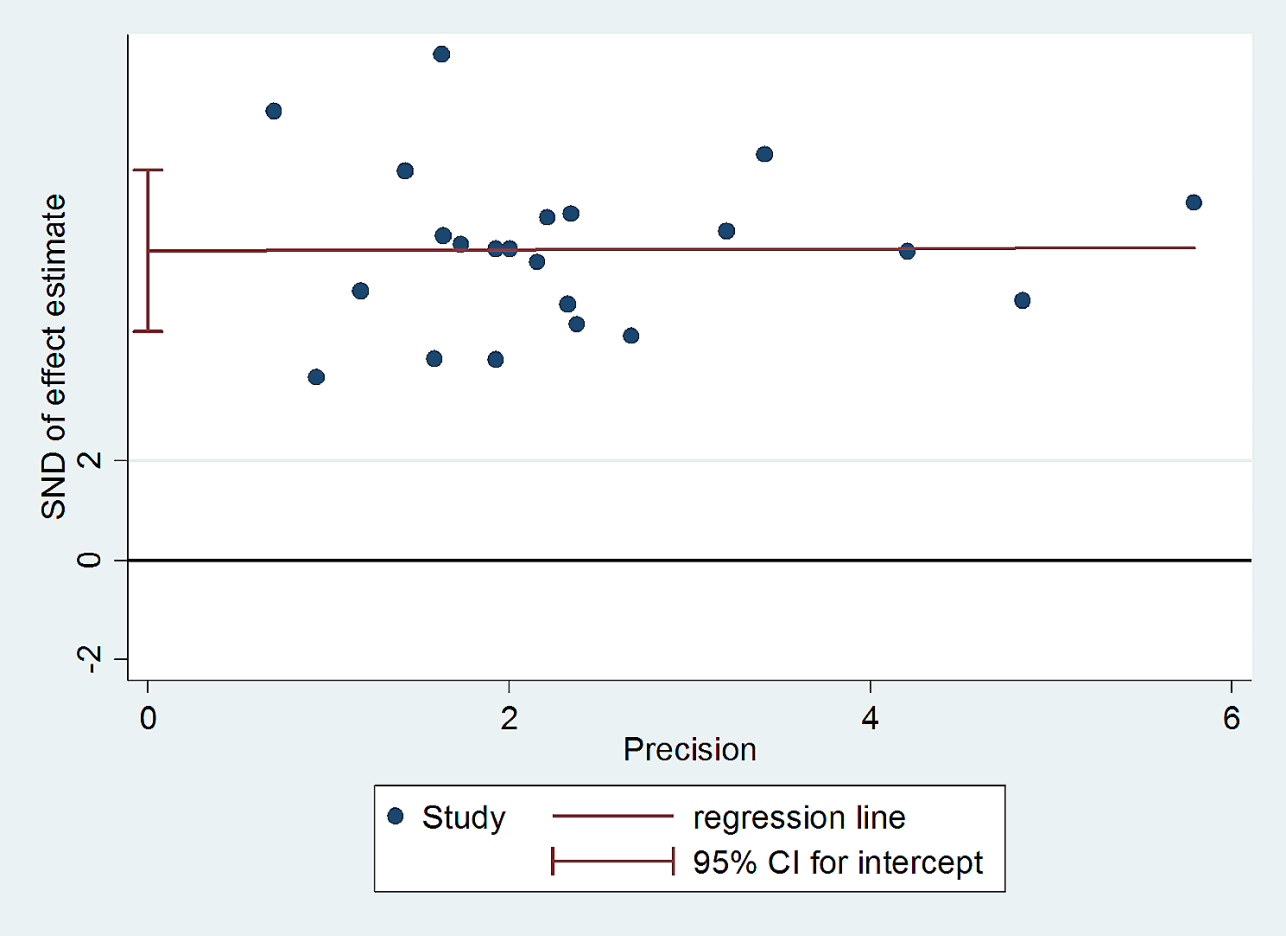
Regression graph of the incidence density of mortality among HIV-infected children after ART initiation in Ethiopia

In addition, as illustrated in Fig. 5 while only four estimates just touched the regression line, the remaining data points were scattered far away from the regression line.

**Fig. 6:**
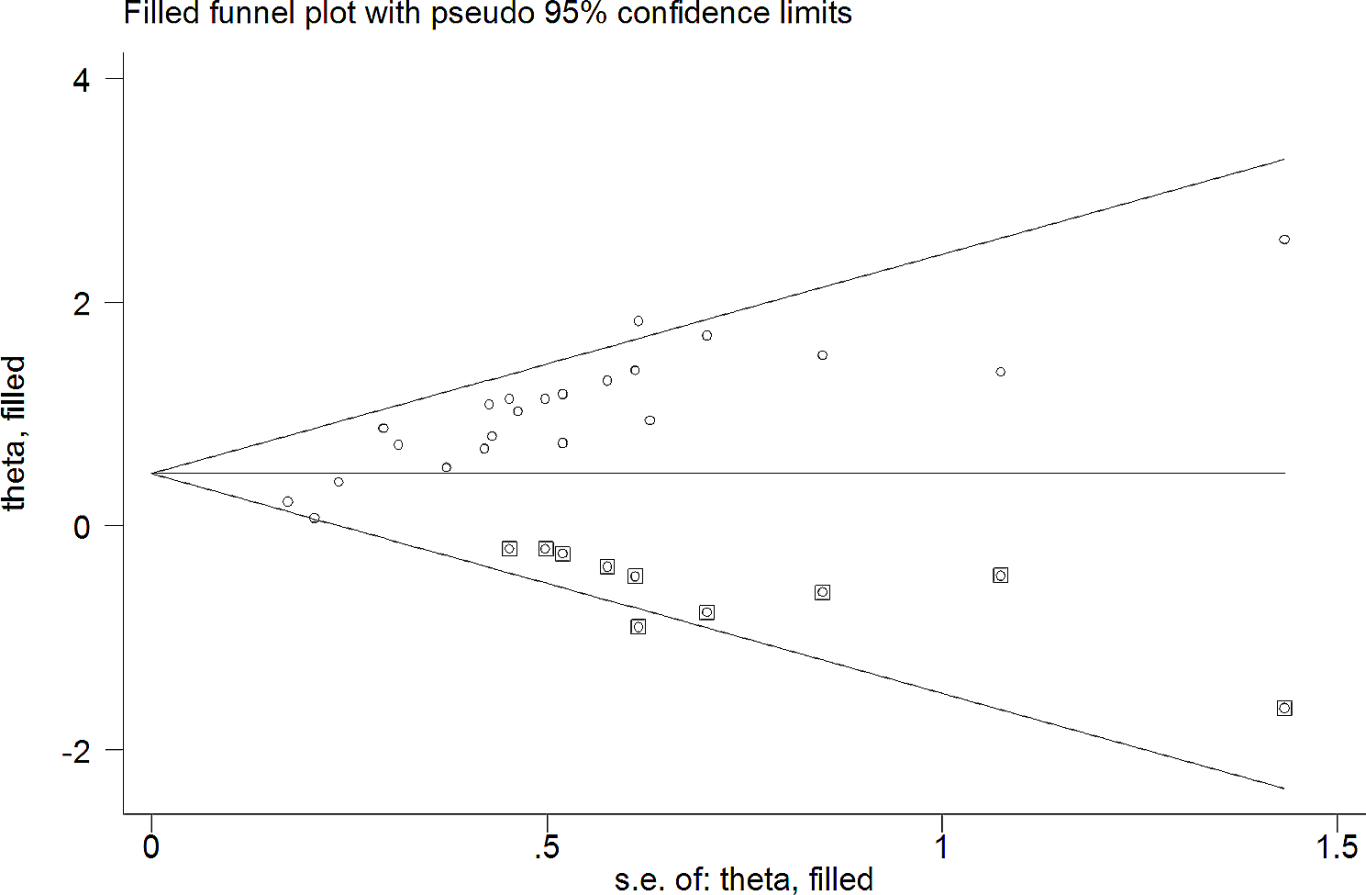
Trim and fill analysis for incidence density of mortality among HIV infected children after ART initiation in Ethiopia

Furthermore, the nonparametric trim-and-fill method of Duval and Tweedie, tests for funnel-plot asymmetry, which provides a way to assess the impact of missing studies because of publication bias on the meta-analysis, was performed. Thus, the meta-trim analysis demonstrated the presence of 10 unpublished studies (Fig. 6) and the filled meta-analysis revealed the pooled incidence density of mortality was 1.83 (95% CI, 1.22 to2.43) per 100 child years.

### Predictors of mortality after antiretroviral therapy initiation

In this systematic review and meta-analysis, being a rural resident, having poor ART adherence, failure to initiate CPT, having low hemoglobin, the presence of OIs, undernutrition (underweight, wasting, and stunting), NVP-based regimens and advanced WHO classified HIV clinical stage were predictors of mortality. The baseline CD4 + T lymphocyte count, IPT, and history of treatment failure, on the other hand, were not significant.

To begin with, the hazards of mortality were 2.18 times (HR, 2.18 [95% CI, 1.20 to 3.98]; *I*^*2*^ = 88.3%) higher among children living in rural settings than among urban residents. The hazard of mortality was 2.85 folds (HR, 2.85 [95% CI, 1.39 to 5.88]; *I*^*2*^ = 93.8%) higher among children poorly adhered to ART. Children who did not take CPT had 2.16 times (HR, 2.16 [ 95% CI, 1.52 to 3.07]; *I*^*2*^ = 81.1%) higher hazards of mortality than those who received prophylactic chemotherapy.

Children with anemia (hemoglobin < 10 g/dl) had 2.28 times (HR, 2.28 [95% CI, 1.51 to 3.45]; *I*^*2*^ = 94.4%) higher hazards of mortality compared to those who had normal baseline hemoglobin levels. The hazards of mortality were 52% higher (HR, 1.52 [95% CI, 1.15 to 2.00]; *I*^*2*^ = 92.9%) among children diagnosed with any of OIs than those who did not exhibit the condition. Underweight, wasted, and stunted children had 74% higher (HR, 1.74 [95% CI, 1.26 to 2.41]; *I*^*2*^ = 91.6%), 2.54 times higher (HR, 2.54 [95% CI, 1.56 to 4.16]; *I*^*2*^ = 82.6%), and 2.02 times higher (HR, 2.02 [95% CI, 1.63 to 2.51]; *I*^*2*^ = 0.0%) hazards of mortality compared to well-nourished children, respectively. Children with advanced WHO clinical stages at presentation (III and IV) had a 71% higher (HR, 1.71 [ 95% CI, 1.42 to 2.05]; *I*^*2*^ = 83.5%) hazard of mortality than those with mild or asymptomatic stages. Lastly, children receiving NVP-based regimens had 3.91 times (HR, 3.91 [95% CI, 3.09 to 4.95]; *I*^*2*^ = 89.9%) (Table 3).

**Table 3:** Meta-analysis of predictor of the mortality among HIV-infected children after ART initiation

Predictors	Citations	Number of studies	Pooled hazard ratio (95% CI)	*P*. value	Heterogeneity		Egger’s test
					I^2^	*P*. value	
Place of residence (rural vs. urban)	[18, 21, 29, 30]	4	2.18 (1.20, 3.98)	0.011*	88.3%	< 0.001	0.961
Adherence to ART (poor vs. good and fair )	[7, 18, 20, 24, 28, 30–32]	8	2.85 (1.39, 5.88)	0.004*	93.8%	< 0.001	0.041
Co-trimoxazole preventive therapy ( no vs. yes)	[18, 20, 24, 26, 30–32]	7	2.16 (1.52, 3.07)	< 0.001*	81.1%	< 0.001	0.012
Isoniazid preventive therapy (no vs. yes)	[18, 20, 24, 27, 30]	5	1.54 ( 0.90, 2.65)	0.115	96.0%	0.115	0.477
Hemoglobin (< 10 g/dl vs. ≥10 g/dl)	[7, 18, 20, 21, 24–27, 29–32, 35]	13	2.28 (1.51, 3.45)	< 0.001*	94.4%	< 0.001	0.001
CD4 + T lymphocyte count (below threshold vs. normal)	[18, 21, 25, 26, 29, 31, 32]	7	1.41 ( 0.84, 2.39)	0.199	96.0%	< 0.001	0.222
Opportunistic infections (yes vs. no)	[7, 18, 21, 25, 29, 31, 32, 34]	8	1.52 ( 1.15, 2.00)	0.003*	92.9%	< 0.001	0.211
Weight for age (underweight vs. normal )	[7, 24, 25, 29, 31]	5	1.74 (1.26, 2.41)	0.001*	91.6%	< 0.001	0.853
Weight for height (wasted vs. normal)	[21, 24, 29, 31]	4	2.54 (1.56, 4.16)	< 0.001*	82.6%	0.001	0.410
Height for age (stunted vs. normal)	[21, 31]	2	2.02 (1.63, 2.51)	< 0.001*	0.0%	0.513	-
WHO classified HIV clinical stages (III and IV vs. I and II)	[7, 21, 23–26, 30, 32, 33, 35]	10	1.71 ( 1.42, 2.05)	< 0.001*	83.5%	< 0.001	0.989
Treatment failure (yes vs. no)	[18, 27, 29]	3	0.34 (0.02, 7.90)	0.505	98.5%	< 0.001	0.754
Nevirapine -based regimens (yes vs. no)	[8, 20, 30, 34]	4	3.91 (3.09, 4.95)	< 0.001*	89.9%	< 0.001	0.003

*, Statistically significant variables at *P*.value ≤ 0.05

## Discussion

Data extracted from 22 studies involving 8,731 children were used to estimate the survival of children after ART initiation. During 31,713.37 person-years of observation, 875 deaths were recorded, with a cumulative incidence of 10%. The weighted random effect meta-analysis showed that the pooled incidence density of mortality among HIV-infected children receiving ART was 3.08 (95% CI, 2.52 to 3.64) per 100 child years of observation. This result was consistent with studies in Nigeria (3 per 100 child years) [35], and India (3 per 100 child years ) [36]. However, our findings were considerably lower than those reported from other African countries: the Democratic Republic of the Congo (3.2 per 100 child years) [37], Malawi (3.4 per 100 child years) [38], South Africa (4.7 per 100 child years) [39], and Kenya (8.4 per 100 child years) [40]. In contrast, it is higher than a study in the United States of America (0.93 per 100 child years) [41], Thailand (1.3 per 100 child years ) [42], rural Zambia (2.0 per 100 child years) [43], Therapeutics Research, Education, and AIDS Training in Asia Pediatric HIV Observational Database study (2.1 per 100 child years ) [44], China (2.3 per 100 child years ), and the United Kingdom and Ireland (2.5 per 100 child years) [45]. Significant variations in the magnitude of mortality can be justified by study size, geographic variations, quality of healthcare facilities, and level of engagement and commitment to implement health policy.

In our study, demographic, clinical, laboratory, and treatment-related predictors of mortality among HIV-infected children after initiation of ART included rural residence, poor adherence to ART, not initiating CPT, low hemoglobin, the presence of OIs, undernutrition (underweight, and wasting), and an advanced WHO HIV clinical stage. Children infected with HIV live in both rural and urban areas. In this meta-analysis, HIV-infected children living in rural settings had twice as high a hazard of mortality after initiation of ART compared to children living in urban areas. This finding is supported by a meta-analysis that investigated the association between place of residence and adherence to ART [46]. The increased death rate among rural residents can be explained by geographic inaccessibility, late presentation to health facilities, and low retention in HIV care and treatment programs.

The hazards of mortality were approximately three-fold greater among children who had poor adherence compared to those who had good or fair adherence to ART. This finding is consistent with a study conducted in India [47], and a cohort study in five countries in the Asia- Pacific region [48]. Poor drug adherence is associated with sustained viral replication, immunological impairment, an increased risk of developing antiretroviral drug resistance, rapid progression of the clinical condition of the child, and decreased survival. Good adherence to ART and decreasing child mortality require the commitment and involvement of responsible caregivers, healthcare providers, social workers, and program managers.

This study also found a two-fold elevated risk of mortality among HIV-infected children who did not receive CPT compared to their counterparts. This finding is consistent with a double-blinded randomized controlled trial conducted among HIV-infected Zambian children receiving ART, where 42% of the placebo group and 28% of the CPT group died during follow-up [49]. Other studies support this finding [39, 50]. Possible justifications include the efficacy of co-trimoxazole against life-threatening infections, such as cerebral toxoplasmosis, puemocystis jirovecii pneumonia, malarial parasitemia, bacterial pneumonia and severe sepsis, which have been described as strong predictors of morbidity and mortality in the HIV -infected population [51–54]. As such, the existing national guidelines for comprehensive HIV prevention, care, and treatment recommend the implementation of CPT as an integral component of a package of HIV-related services and a general strategy to prevent OIs [55].

Anemia was another predictor variable identified in our analysis, and the hazards of mortality were 2.28-fold greater among HIV-infected children with baseline hemoglobin < 10 g/dl compared to those with hemoglobin 10 g/dl and above. This finding was supported by studies conducted in Malawi [56], Tanzania [57], Kenya [40], and a multicenter study in Europe [58]. Moreover, a systematic review and meta-analysis by Wubneh et *al* [59], described that HIV-infected children with baseline hemoglobin < 10 g/dl had a 2.42 times higher risk of death. Anemia is common throughout HIV infection and may be the direct result of HIV infection and harbors underlying opportunistic neoplasms such as lymphoma, OIs such as systemic fungal and mycobacterium infections, and nutritional deficiencies. In addition, antiretroviral and other medication toxicitiesare associated with bone marrow suppression, resulting in leukopenia, thrombocytopenia, and anemia, further challenging the survival of HIV-infected children while receiving ART. Anemia prevalence rates of 39.7% [60] and 22.3% [61] have been observed globally and in Ethiopia, respectively, among HIV-infected children after ART initiation.

This study also revealed that HIV-infected children who exhibited OIs had a 52% higher risk of mortality after ART initiation than their counterparts. Similarly, research supporting the current finding was reported in Tanzania, which stated that children diagnosed with OIs had greater mortality hazards [57]. Moreover, OIs such as chronic diarrhea were found to be important predictors of mortality among HIV-infected children, in a South African study [39]. In Ethiopia, although ART is initiated for all HIV-infected patients as rapidly as possible, irrespective of their immunological status, up to 53% of patients present for care and treatment at late clinical stages with acquired immune deficiency syndrome -defining OIs [62].

In agreement with other studies conducted elsewhere [40, 57], the mortality hazards among underweight, wasted, and stunted children were 74% higher, three times, and twice that of well-nourished children, respectively. The possible justification emanates from the synergistic effect of both HIV and undernutrition; HIV causes poor appetite secondary to chronic inflammation, and enteropathy interferes with nutrient absorption from the gastrointestinal tract [63]. Undernutrition, in turn, accelerates the progression of the clinical stages of HIV because of the direct effect of undernutrition on immunity, all of which negatively affect the survival of HIV-infected children [64, 65].

Predictor analysis also found that children receiving NVP-based regimens had an estimated four times greater risk of mortality compared to those not receiving NVP-based regimens. Research supporting the current finding was reported in low-resource settings [66–68]. Possible justifications include life-threatening hepatotoxicity and severe skin rashes associated with the nonnucleoside reverse transcriptase inhibitor, NVP.

Finally, findings of this systematic review and meta-analysis revealed that children who presented with advanced diseases (WHO clinical stages II and IV) to chronic HIV care and treatment had 71% higher hazards of mortality compared to those children with mild diseases (WHO classified HIV clinical stages I and II). Other studies found similar results to ours [41, 57, 69–71]. This is because life-threatening OIs and malignancies occur at the advanced HIV clinical stages and remain the major drivers of HIV-related mortality and morbidity.

### Strengths and limitations of this study

To the best of our knowledge, this systematic review and meta-analysis is the first of its kind to investigate the magnitude of mortality and its predictors among HIV-infected children receiving ART in Ethiopia, and with strong conviction, the findings of this meta-analysis contribute to the provision of comprehensive HIV care and treatment for HIV-infected children in resource limited regions including Ethiopia. Furthermore, evidence can be utilized by researchers, policymakers, clinicians, and other stakeholders in resource-poor settings. This study had some limitations. First, significant statistical heterogeneity was observed for the pooled mortality estimate. As the qualities of retrospective cohort studies were not as high as randomized controlled trial and prospective studies, the study design may be one of the main source of heterogeneity. Therefore, this requires a cautious interpretation of the results. Second, only articles published in English were included.

## Conclusions and recommendations

In conclusion, the pooled incidence density of mortality among HIV-infected children receiving ART was 3.08 per 100 child years in Ethiopia. Predictors of mortality included being a rural resident, poor ART adherence, failure to initiate CPT, anemia, the presence of OIs, undernutrition (underweight, wasting, and stunting), NVP-based regimens and an advanced WHO clinical stage at presentation to an HIV care and treatment center. Therefore, high-level commitment and involvement of responsible caregivers, healthcare providers, social workers, and program managers are required to strengthen and promote good ART adherence. Furthermore, the screening and management of anemia, undernutrition, and OIs according to the national ART guidelines are of paramount importance to enhance the survival of HIV-infected children receiving ART.

### Electronic supplementary material

Below is the link to the electronic supplementary material.

Supplementary Material 1

Supplementary Material 2

Supplementary Material 3

Supplementary Material 4

## Data Availability

The dataset supporting the conclusions of this article are included within the article and its additional files.

## Abbreviations

ART: Antiretroviral therapy
CD4: Cluster of differentiation 4
CI: Confidence interval
CPT: Cotrimoxazole preventive therapy
HIV: Human immune deficiency virus
HR: Hazard ratio
IPT: Isoniazid preventive therapy
NVP: Nevirapine
OIs: Opportunistic infections
PRISMA: The Prospective Reporting Items for Systematic Reviews and Meta-Analysis
SNNPR: The Southern Nations, Nationalities, and Peoples Region
WHO: The World Health Organization
