# Supplementary material for: Mortality and its predictors among human immunodeficiency virus-infected children younger than 15 years receiving antiretroviral therapy in Ethiopia: a systematic review and meta-analysis
Source: BMC Infect Dis. 2024 May 3;24:471. doi: 10.1186/s12879-024-09366-1 (PMC11069260; doi:10.1186/s12879-024-09366-1)
Supplement: Supplementary file 2 — Supplementary Material 2 [file 12879_2024_9366_MOESM2_ESM.docx]

S1 table : search details

| Search terms | Query |
| --- | --- |
| “ mortality”, “death”, “survival”, “HIV/AIDS”, “Human immune deficiency virus”, “acquired immune deficiency syndrome”, “ART”, “antiretroviral therapy”, “HAART”, “highly active antiretroviral therapy”, “prevalence,” “proportion”, “incidence”, “associated factors”, “predictors”, “determinants”, “child*”, “pediatrics”, “paediatrics”, “Ethiopia” | mortality OR death OR survival AND HIV/AIDS OR Human immune deficiency virus OR acquired immune deficiency syndrome AND ART OR antiretroviral therapy OR HAART OR highly active antiretroviral therapy AND prevalence OR proportion OR incidence AND associated factors OR predictors OR determinants AND child* OR pediatrics OR paediatrics AND Ethiopia AND (english[Filter]) |

8271
