# Supplementary material for: Mortality and its predictors among human immunodeficiency virus-infected children younger than 15 years receiving antiretroviral therapy in Ethiopia: a systematic review and meta-analysis
Source: BMC Infect Dis. 2024 May 3;24:471. doi: 10.1186/s12879-024-09366-1 (PMC11069260; doi:10.1186/s12879-024-09366-1)
Supplement: Supplementary file 3 — Supplementary Material 3 [file 12879_2024_9366_MOESM3_ESM.docx]

**S2Table**  : Quality assessment of studies using JBI’s critical appraisal tools designed for Cohort studies

| Study | JBI’s critical appraisal questions  Score | | | | | | | | | | | | Score | Overall Appraisal |
| --- | --- | --- | --- | --- | --- | --- | --- | --- | --- | --- | --- | --- | --- | --- |
|  | Q1 | Q2 | Q3 | Q4 | Q5 | | Q6 | Q7 | Q8 | Q9 | Q10 | Q 11 |  |  |
| Mulugeta, 2017 [8] | Y | Y | Y | Y | | U | Y | Y | Y | Y | Y | Y | 10 | Included |
| Gesesew,2018 [9] | Y | Y | Y | Y | | Y | Y | Y | U | Y | Y | Y | 10 | Included |
| Edessa,2016 [10] | Y | Y | Y | Y | | Y | Y | Y | Y | Y | Y | Y | 11 | Included |
| Chanie, 2021 [11] | Y | Y | Y | Y | | Y | Y | Y | N | Y | Y | Y | 10 | Included |
| Kedir,2014 [12] | Y | Y | Y | Y | | Y | Y | Y | Y | Y | Y | Y | 11 | Included |
| Atnafu, 2012 [13] | Y | Y | Y | Y | | Y | Y | Y | Y | Y | Y | Y | 11 | Included |
| Dawit, 2021 [14] | Y | Y | Y | N | | Y | Y | Y | Y | Y | Y | Y | 11 | Included |
| Alebel,2020 [15] | Y | Y | Y | Y | | y | N | Y | Y | Y | Y | Y | 10 | Included |
| Asfawesen, 2011 [16] | Y | Y | Y | Y | | Y | N | Y | N | Y | Y | Y | 10 | Included |
| Ebissa, 2015 [17] | Y | Y | Y | Y | | Y | Y | Y | Y | N | N | N | 9 | Included |
| Nigussie, 2022 [18] | Y | Y | Y | Y | | Y | Y | Y | N | Y | Y | Y | 10 | Included |
| Koye,2012 [19] | Y | Y | Y | Y | | Y | Y | Y | N | Y | Y | Y | 10 | Included |
| Gebremedhin,2013 [20] | Y | Y | Y | Y | | Y | Y | Y | N | Y | Y | Y | 10 | Included |
| Gemechu, 2022 [21] | Y | Y | Y | Y | | Y | Y | Y | N | Y | Y | Y | 10 | Included |
| Tagesse, 2020 [22] | Y | Y | Y | Y | | Y | Y | Y | N | Y | Y | Y | 10 | Included |
| Bitew, 2017 [23] | Y | Y | Y | Y | | Y | Y | Y | N | Y | Y | Y | 11 | Included |
| Atalell, 2018 [24] | Y | Y | Y | Y | | Y | Y | Y | N | Y | Y | Y | 10 | Included |
| Biyazin, 2022 [25] | Y | Y | Y | Y | | Y | Y | Y | N | Y | Y | Y | 10 | Included |
| Arage, 2019 [26] | Y | Y | Y | Y | | Y | Y | Y | N | Y | Y | Y | 10 | Included |
| Chekole, 2022 [27] | Y | Y | Y | Y | | Y | Y | Y | N | Y | Y | Y | 10 | Included |
| Sidamo, 2018 [28] | Y | Y | Y | Y | | Y | Y | Y | N | Y | Y | Y | 10 | Included |
| Andargie, 2018 [29] | Y | Y | Y | Y | | Y | Y | Y | N | Y | Y | Y | 10 | Included |

Y –Yes;N-No;U -Unclear-Question. Overall score is calculated by counting the number of Y’s in each row. Q1)Were the two groups similar and recruited from the same population?Q2) Were the exposures measured similarly to assign people to both exposed and unexposed groups? Q3) Was the exposure measured validly and reliably? Q4) Were confounding factors identified? Q5) Were strategies to deal with confounding factors stated? Q 6) Were the groups/participants free of the outcome at the start of the study (or at the moment of exposure)? Q7)Were the outcomes measured validly and reliably? Q8)Was the follow-up time reported sufficient to be long enough for outcomes to occur? Q9)Was the follow-up complete, and if not, were the reasons for the loss to follow-up described and explored? Q10)Were strategies to address incomplete follow-up utilized?, Q11) Was appropriate statistical analysis used?
